# Abnormal outer and inner retina in a mouse model of Huntington’s disease with age

**DOI:** 10.3389/fnagi.2024.1434551

**Published:** 2024-10-28

**Authors:** Dashuang Yang, Chunhui Huang, Xuemeng Guo, Yintian Li, Jiaxi Wu, Zaijun Zhang, Sen Yan, Ying Xu

**Affiliations:** ^1^Guangdong-Hongkong-Macau Institute of CNS Regeneration, Key Laboratory of CNS Regeneration (Jinan University)-Ministry of Education, Guangdong Key Laboratory of Non-human Primate Research, Guangzhou, China; ^2^School of Medicine, Jinan University, Guangzhou, China; ^3^State Key Laboratory of Bioactive Molecules and Druggability Assessment, and Guangzhou Key Laboratory of Innovative Chemical Drug Research in Cardio-cerebrovascular Diseases, and Institute of New Drug Research, Jinan University, Guangzhou, China; ^4^Guangdong-Hong Kong-Macau Joint Laboratory for Pharmacodynamic Constituents of TCM and New Drugs Research, and Guangdong Province Key Laboratory of Pharmacodynamic Constituents of TCM and New Drugs Research, Jinan University College of Pharmacy, Guangzhou, China; ^5^International Cooperative Laboratory of Traditional Chinese Medicine Modernization and Innovative Drug Development of Chinese Ministry of Education (MOE), Jinan University College of Pharmacy, Guangzhou, China; ^6^Co-Innovation Center of Neuroregeneration, Nantong University, Nantong, China

**Keywords:** Huntington’s disease, R6/1, mutant huntingtin, retina, photoreceptor, ganglion cell, bipolar cell, optic nerve

## Abstract

Huntington’s disease (HD) is a progressive neurodegenerative disorder characterized by motor dysfunction and cognitive decline. While retinal abnormalities have been documented in some HD patients and animal models, the nature of these abnormalities—specifically whether they originate in the inner or outer retina—remains unclear, particularly regarding their progression with age. This study investigates the retinal structure and function in HD transgenic mice (R6/1) compared to C57BL/6 J control mice at 2, 4, and 6 months of age, encompassing both pre-symptomatic and symptomatic stages of HD. Pathological assessments of the striatum and evaluations of motor function confirmed significant HD-related alterations in R6/1 mice at 6 months. Visual function was subsequently analyzed, accompanied by immunofluorescent staining of retinal and optic nerve tissues over time. Our findings revealed that R6/1 mice exhibited pronounced HD symptoms at 6 months, characterized by neuronal loss in the striatum and impaired locomotor abilities. Functionally, visual acuity declined at 6 months, while retinal light responses began to deteriorate by 4 months. Structurally, R6/1 mice demonstrated a global reduction in cone opsin expression as early as 2 months, with a decrease in rhodopsin levels at 4 months, alongside a thinner retinal structure compared to controls. Notably, rod bipolar cell populations were decreased at 6 months, exhibiting shorter dendritic branches and reduced synaptic connections with photoreceptors in the outer retina. Additionally, ganglion cell numbers in the inner retina decreased at 6 months, accompanied by aberrant neural fibers in the optic nerve. Microglial activation was evident at 4 months, while astrocytic activation was observed at 6 months. Aggregates of mutant huntingtin (mHTT) were first detected in the ganglion cell layer and optic nerve at 2 months, subsequently disseminating throughout all retinal layers with advancing age. These results indicate that retinal pathology in R6/1 mice manifests earlier in the outer retina than in the inner retina, which does not align with the progression of mHTT aggregation. Consequently, the R6/1 mouse retina may serve as a more effective model for elucidating the mechanisms underlying HD and evaluating potential therapeutic strategies, rather than functioning as an early diagnostic tool for the disease.

## 1 Introduction

Huntington’s disease (HD) is a chronic, progressive neurological disorder characterized by the degeneration and loss of neurons, ultimately leading to mood disturbances, cognitive decline, and involuntary movements as neurons deteriorate (Schiefer et al., 2015). This disease arises due to an abnormal expansion of cytosine-adenine-guanine (CAG) repeats, which translates into an elongated glutamine (Gln, Q) repeat in the Huntingtin protein. This mutation triggers pathological alterations, including striatal neuronal degeneration, cortical atrophy, and ventricular system enlargement (Jimenez-Sanchez et al., 2017; Jia et al., 2023).

With the progression of therapeutic interventions for HD disease and other neurodegenerative diseases such as Alzheimer’s disease (AD) (Klyucherev et al., 2022), Parkinson’s disease (PD) (Alves et al., 2023), and amyotrophic lateral sclerosis (ALS) (Rojas et al., 2020; Soldatov et al., 2021), the retina has emerged as a crucial diagnostic tool that objectively monitor disease progression and evaluate treatment efficacy. Clinically, HD patients have exhibited segmental thinning of the retinal nerve fiber layer (RNFL) around the optic disc and a reduction in macular volume during the onset of the disease (Gulmez Sevim et al., 2019; Dusek et al., 2023), along with a notable impaired color vision (Gulmez Sevim et al., 2019). These observations suggest a substantial impact of HD on the retina. Furthermore, studies have reported reduced light responses from both the retina and visual cortex in HD patients (Ellenberger et al., 1978; Oepen et al., 1981; Josiassen et al., 1984; Hennerici et al., 1985; Dhalla et al., 2019). However, research findings on the specific impact of HD on inner versus outer retinal pathology have been inconsistent. Some studies have reported thinning of retinal layers, notably the RNFL (Gulmez Sevim et al., 2019), whereas others have not (Dusek et al., 2023). Similarly, while outer retinal dysfunction has been reported in HD patients, inner retinal dysfunction has not been uniformly observed. Additionally, correlations between disease progression and retinopathy in HD patients have also varied.

Animal models of HD have been instrumental in validating retinopathy observed in HD patients. Researchers have employed R6/1 and R6/2 transgenic mice, carrying mutations in the human HTT gene that lead to slow and rapid progression of HD symptoms, respectively, to study HD-associated retinal pathology. In these mice models, gradual alterations in the outer retina, particularly affecting the cones, were observed. These changes were accompanied by the impaired cone and bipolar responses, as evidenced by electroretinogram (ERG) recording (Helmlinger et al., 2002; Batcha et al., 2012). Additionally, reactive gliosis, indicative of retinal inflammation, was reported in R6/1 at various stages of HD progression (Batcha et al., 2012; Cano-Cano et al., 2024). Recently, a thinning of myelin sheaths in the optic nerve was reported in symptomatic R6/2 mice (Mazur-Michałek et al., 2022), suggesting the impairment of retinal ganglion cells, whose axons form the optic nerve. However, the inner retinal ganglion cells appear morphologically normal (Ragauskas et al., 2014) and the progression of the disease within the retina remains elusive. Further clarification is needed regarding the age-related pathological changes in both the outer and inner retina of HD mouse models. Moreover, the correlation between retinopathy progression and the accumulation of mutant huntingtin protein (mHTT) is yet to be determined.

In the current study, we aimed to investigate whether R6/1 transgenic mice exhibit retinopathy in both the inner and outer retina, and to explore how this retinopathy progresses with age. Furthermore, we sought to determine the correlation between retinopathy and the accumulation of mHTT. Our objective is to establish whether the retina of an HD mouse can serve as a valid clinical model for studying HD pathology.

## 2 Materials and methods

### 2.1 Animals

The B6.Cg-Tg (HDexon1) 61Gpb/J mice (R6/1 mice) were purchased from the Jackson Laboratory (Bar Harbor, ME, USA). The R6/1 strain carries 116–150 CAG repeats in exon 1 of the human HTT gene, and it is a single copy integrant (Mangiarini et al., 1996). The offspring were obtained by mating male R6/1 mice with female C57BL/6 mice. The mice were then identified by PCR: if a 170 bp band was obtained in the PCR product (primer sequence 5′-3′: CCG CTC AGG TTC TGC TTT TA and TGG AAG GAC TTG AGG GAC TC), the mice were R6/1 positive. The litter-mates that showed negative for the band were taken as wild type (WT) control. All animals were housed 3–5 per cage with *ad libitum* access to water and food during a 12-h light/dark cycle under controlled temperature (23 ± 2°C) and humidity (40–60%). Use and care of mice followed the guidelines of the Institutional Animal Care and Use Committee of Jinan University (Guangzhou, China), license number: SYXK (Guangdong) 2012–0117. All operations in the experiment followed the regulations of the NIH and the Experimental Animal Ethics Committee of Jinan University. As there is no report on the gender differences in either the motor or visual abnormalities of R6/1, both female and male mice were tested. The detailed gender of animal used for each test is listed in Supplementary Table S1.

### 2.2 Rotarod test

The rotarod test was used to assess the motor coordination of mice in a rotating state. The rod was a knurled plastic dowel (6.0 cm diameter) set at a height of 30 cm. Mice were trained 3 days to stay on the rod rotating at a speed of 10 rpm/s for 10 min before testing. The time of the mouse falling from the rod was automatically recorded by a computer. After training, each mouse was tested 3 times on the accelerating rotarod (30 rpm) for 5 min. The time to fall during each trial was recorded and averaged.

### 2.3 Balance beam test

The balance beam test consists of an elevated and narrow beam (1 cm wide and 80 cm long) that allowed the mice to trespass to reach a safe area. The time for the mouse to cross the balance beam was recorded. Before the test, mice were trained for 3 days to cross the beam. After training, each mouse was tested 3 times and the time to cross the beam was recorded.

### 2.4 Dark–light transition test

To assess the visual ability of the mouse in detecting luminance, a dark–light transition test was conducted as we previously described (Zhang et al., 2017). The tests were conducted between 9 a.m. and 6 p.m. during the day. The system consisted of a light-illuminated chamber and a dark chamber with open access that allowed the mice to travel freely between them. For each mouse, the movement was recorded by a camera and the time that a mouse spent in the black chamber during a five-minute trial was recorded using the EthoVision XT 8.0 software (Noldus). Mouse are nocturnal and prefer darkness, so for visually normal mice, the time spent in the dark box would be longer than in the light room. But for the visually impaired mouse, as it lost the ability to tell luminance, the time spent in the two chambers would be similar. Therefore, a longer time spent in the dark box indicates a better visual ability of the animal.

### 2.5 Optokinetic response test (optomotor)

The optomotor test was employed to assess the visual ability of the mouse to tell objects with fine spatial frequency as we previously described (Kong et al., 2024). In brief, after an overnight dark adaption, the 6-month-old mouse was placed upon a platform encompassed by four computer screens, which displayed moving vertical sine gratings generated by a program written with MATLAB software. The gratings with increasing spatial frequencies (including 0.1, 0.2, 0.3, 0.35, 0.4, 0.45, and 0.5 cycles/degree, 100% contrast) were displayed for 60 s in both clockwise and counterclockwise directions. For each frequency, the head movements of the mouse were recorded with a video camera. A head rotation following the direction of the moving grating was considered a positive optokinetic response. The highest spatial frequency at which an optokinetic response was evoked was recorded as the visual acuity of the responsive eye.

### 2.6 Electroretinogram (ERG) recording

Mice were dark-adapted overnight and the ERG was measured with a RETI-scan system (Roland Consult, RETI-scan, Germany) as previously described by us (Yang et al., 2015). Briefly, mice were anesthetized with tribromoethanol (0.14 L/10 mg bodyweight of 1.25% solution) and placed on a heated platform (37°C) under dim red light. Pupils were dilated with phenylephrine HCl (0.5%) and tropicamide (0.5%). ERGs were recorded with gold-plated wire loop electrodes contacting the corneal surface as the active electrode. Stainless steel needle electrodes were inserted in the skin near the eye and in the tail serving as reference and ground leads, respectively. Following adaptation, animals were stimulated with green flashes of graded intensities of 0.01, 0.1, and 3.0 cd.s/m^2^, then light adapted for 5 min under a bright green background (20 cd/m^2^) before recording photopic responses to green flashes of 3.0 and 10.0 cd.s/m^2^. The ERG data were collected via the amplifier of RETI-scan system at a sampling rate of 2 kHz and analyzed with the RETIport software (Roland) after 50 Hz low-pass filtering. The a-wave amplitude was measured from the baseline to the first negative peak, and the b-wave amplitude was measured from a-wave trough to the next positive peak. For each animal, the best response of the two eyes was taken as one data point.

### 2.7 Western-blotting

After anesthetizing the mice with isoflurane, the mice were intracardially perfused with saline. Striatal tissues of mice were subsequently obtained and were homogenized with ice-cold RIPA lysis buffer (10 mM Tris, pH 7.4, 100 mM NaCl, 1 mM EDTA, 1 mM EGTA, 0.1% SDS, 1% Triton X-100) containing protease inhibitors. The lysates were sonicated for 10 s and incubated on ice for 30 min and then centrifuged at 12,000 × g for 3 min at 4°C. The total protein concentration of the supernatant was determined using the Bradford method (BCA protein assay kit, Pierce, Rockford, IL). Samples containing 25 μg of protein were separated by 10–15% (w/v) SDS-PAGE and transferred to a methanol-activated NC membrane. Then, the membrane was blocked in 5% (w/v) bovine serum albumin in TBST at room temperature for 1 h and subsequently probed with primary antibody. The antibody information is listed in Table 1. After incubated with HRP-conjugated secondary antibody in TBST for 1 h at room temperature (1:5,000, Cell Signaling Technology, Danvers, MA, USA), the reaction product in the membrane was visualized using an ECL advanced Western blot detection kit and chemical imaging system (Clinx, Shanghai, Shanghai). Western blot images and the densities of the bands were captured and quantitatively assessed using the Image J Software.

**Table 1:** Information of antibodies used in Western blotting.

Antibody/stain	Source	Catalog #	RRID	Dilution
Mouse monoclonal anti-8G	From Li-lab, Jinan University, China			1:50
Rabbit monoclonal anti-Darpp32	Cell signaling, Danvers, Massachusetts, USA	2306S		1:1000
Rabbit monoclonal anti-Iba1	Abcam, Cambridge, UK	ab178846	AB_2636859	1:1000
Rabbit monoclonal anti-NeuN	Abcam	ab177487	AB_2532109	1:1000
Rat polyclonal anti-GFAP	Abcam	ab7260	AB_305808	1:1000

### 2.8 Tissue collection, immunofluorescent staining, and microscopy

Mice were deeply anesthetized with isoflurane and intracardially perfused with saline. The mouse brains were then soaked in 4% (w/v) paraformaldehyde (PFA) (0.1-M PBS, pH 7.4) for 24 h before dehydration with 30% sucrose solution and cryo-sectioned into 16 μm thick sections. Brain sections were incubated with 0.1% (v/v) Triton X-100 for 15 min before blocking with 10% (v/v) horse serum in PBS (0.01 M, pH 7.4) for 1 h at room temperature. Afterward, brain sections were incubated overnight with primary antibodies. The antibody information is listed in Table 2. Then, the sections were incubated with Alexa Fluor^®^ 488, 594, or 647-conjugated goat anti-rabbit or mouse or rat (1:1,000, Invitrogen, Carlsbad, CA, USA) for 1 h at room temperature. Cell nuclei were counterstained with DAPI for 10 min.

**Table 2:** Information on antibodies used in immunofluorescent staining.

Antibody/stain	Source	Catalog #	RRID	Dilution	Tissue
Primary					
Chicken polyclonal anti-GFAP	Aves Labs, Davis, California, USA	GFAP5727980	AB_2313547	1:1000	Retina
Mouse monoclonal anti-8G	From Li-lab, Jinan University, China			1:50	Brain Retina
Mouse monoclonal anti-PSD95	Millipore, Burlington, MA, USA	MAB1596	AB_2092365	1:500	Retina
Mouse monoclonal anti-Rhodopsin	Millipore	MAB5356	AB_2178961	1:1000	Retina
Rabbit monoclonal anti-Darpp32	Cell signaling, Danvers, Massachusetts, USA	2306S		1:1000	Brain
Rabbit monoclonal anti-Iba1	Abcam, Cambridge, UK	ab178846	AB_2636859	1:1000	Brain
Rabbit monoclonal anti-NEFM	Huabio, Hangzhou, Zhejiang, CHN	ET1703-57	AB_3070413	1:200	Retina
Rabbit monoclonal anti-NeuN	Abcam	ab177487	AB_2532109	1:1000	Brain
Rabbit polyclonal anti-Iba1	FUJIFILM WAKO SHIBAYAGI, Shibayagi, Japan	019–19,741	AB_839504	1:1000	Retina
Rabbit polyclonal anti-Opsin, red/green	Abcam	ab5405	AB_177456	1:1000	Retina
Rabbit polyclonal anti-PKCα	Sigma-Aldrich	P4334	AB_477345	1:10000	Retina
Rabbit polyclonal anti-RBPMS	Novus biologicals	NBP2-20112	AB_3075531	1:1000	Retina
Rat monoclonal anti-GFAP	Invitrogen, Carlsbad, CA, USA	13–0300	AB_86543	1:1000	Brain
Stain					
Donkey DAPI	Electron Microscopy Sciences, Hatfield, PA, USA	17,984–24		1:1000	

To examine the structure of the retina, after sacrificing the animal, whole eye globes were fixed for 30 min in 4% PFA after cornea and iris removal for a good fixation of the retina. After three washes with PBS, eyes were then soaked in 30% sucrose overnight and processed for cyro-sectioning the following day. For sectioning, retinas were frozen in O.C.T and cryosectioned at 16 μm thickness longitudinally through the optic disk. To do the immunofluorescent staining, retinal sections were washed in PBS three times for 5 min and then blocked in blocking buffer containing 10% donkey serum albumin (Solarbio, SL034) in 0.3% PBST with Triton X-100 (Solarbio, #78200) for 2 h at room temperature, then incubated with the primary antibodies overnight in blocking buffer at 4°C. The following day sections were rinsed three times for 5 min in PBS and secondary antibodies were applied at a concentration of 1:1,000 in PBS for 2 h. Slides were washed again in PBS and then coverslipped using an Antifluorescent quencher. For the wholemount retina, 0.3% PBST was replaced with 3% PBST for the blocking buffer, and the incubation time of the primary antibody was extended to 2 nights. The antibody information is listed in Table 2. Among them, the mouse monoclonal anti-8G, also known as mEM48, can specifically recognize mHTT aggregates by binding to ploy Q on the mHTT protein (Yan et al., 2023; Qin et al., 2024). After the incubation with the primary antibody, the sections were incubated with Alexa Fluor^®^ 488, 594, or 647-conjugated donkey anti-rabbit or mouse or chicken (1:1,000, Invitrogen, Carlsbad, CA, USA) for 2 h at room temperature.

### 2.9 Image collection and processing

For the brain tissues, the striatum was viewed with TissueGnostics panoramic tissue and cell quantitative analysis system or the laser scanning confocal microscope (Olympus FV3000 microscope). Three mice per group were used for analysis. For each animal, three images with an image size of 320 × 320 μm were taken, and values were then averaged to get one data point for this animal.

For retinal tissues, the florescent images of the retina were photographed with a fluorescence microscope (Zeiss Axio Imager A2) and confocal microscope (Zeiss LSM700, Oberkochen, Germany). At least three full-length sections from each animal were assessed to generate an average expression pattern of the retina for each animal. Images at 500, 1,000, and 1,500 μm away from the center of the optic disc (OD) center were sampled as the central, middle, and peripheral regions, respectively. To measure the thickness of the retina, lines vertical to the retinal layers were drawn, and the length of the lines was measured by Image J (NIH). To measure the length of bipolar cell dendrites, the longest dendrite in each image field was chosen and measured by ZEN 2.3 (blue edition). To compare the fluorescent intensity, tissues from different animal groups at the same age were processed with the same immunostaining protocol and image parameters. The mean intensity across the region of interest was measured by ZEN and then normalized to the mean value from the center region of control group. ZEN was also applied to count the mHTT number or cell numbers. For the wholemount retina, regions from center (600 μm) to peripheral (1,800 μm from OD) on each of the quadrant pieces were analyzed and averaged. For the optic nerve, regions along the entire length was analyzed at the proximal, middle, and distal end, respectively. For each animal, three to six images per retina with an image size of 320× 320 μm or 160 × 160 μm were taken, and values were then averaged to get one data point for this animal, shown as individual dots on the results plot.

### 2.10 Statistical analysis

All data are expressed as mean ± SEM. Student’s t-test or two-way ANOVA with Sidak’s post-hoc tests was performed with GraphPad 8 (GraphPad Software, San Diego, CA, USA) depending on the number of groups compared. Statistical significance was determined at *p* < 0.05, and *p* < 0.01 was considered highly significant. Unless otherwise stated, the “n” indicates the number of mice examined for each group.

## 3 Results

### 3.1 R6/1 mice demonstrate severe HD symptoms and reduced visual acuity at 6 M

**Figure 1:**
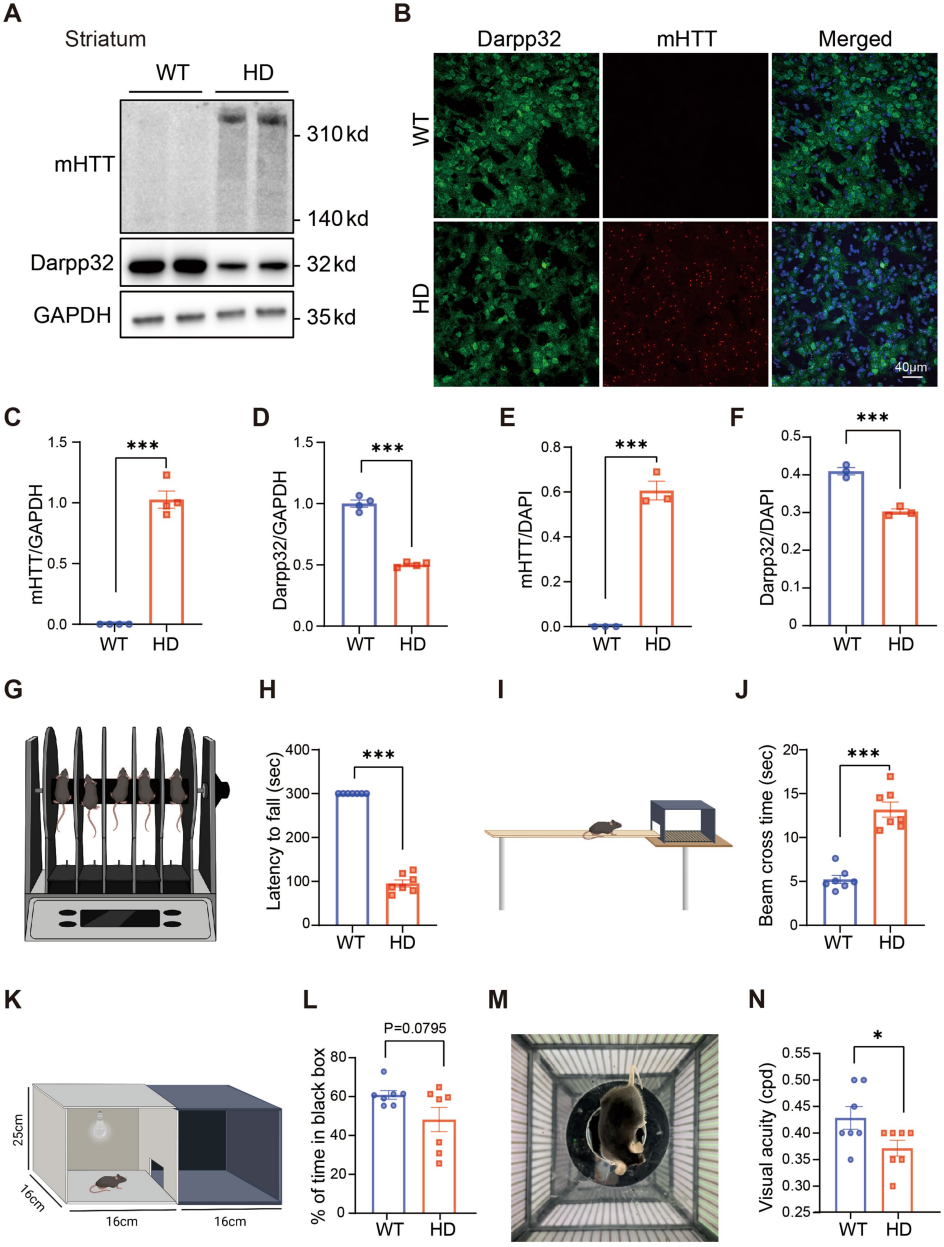
Impaired visual functions in R6/1 mouse at 6 M. **(A)** Western blotting showing the expression of mHTT and Darpp32 in the striatum of 6-month-old R6/1 transgenic mice, with GAPDH as the reference. **(B)** The striatum slice from a WT and an R6/1 mouse at the age of 6 M was stained by anti-8G (red) to label mHTT and anti-Darpp32 (green) to label multi-spiny neurons. DAPI (blue) was also stained to label the nuclei of cells. **(C,D)** Quantitation of the expression of mHTT **(C)** or Darpp32 **(D)** as a ratio to GAPDH. **(E,F)** The ratio of mHTT-positive cell number **(E)** or Darpp32-positive neuron number **(F)** of the total cells (DAPI positive) per image in WT and HD mouse striatum. **(G,I)** Illustration of the rotarod **(G)** and balance beam **(I)** test to test the motor ability. **(H)** The latency to fall from the rotating rod for different animal groups. **(J)** Time to cross the beam in experimental animal groups. HD mice spent a significantly shorter time staying on the rotarod and a longer time crossing the beam than WT. **(K,M)** Illustration of the dark–light transition box **(K)** and optomotor **(M)** system for the visual behavior test. **(L)** The percentage of time at which animals stayed in the dark box (relative to the total time in both boxes) did not differ between WT and HD mice. **(N)** The visual acuity (in cycles per degree, cpd) was lower in HD mice than in the WT control. Data are expressed as mean ± SEM; *p < 0.05, **p < 0.01, ***p < 0.001, student t-test. WT, C57BL/6 J, HD, Huntington’s disease. Each dot in the graph represents the data from one animal.

We first investigated HD symptoms in R6/1 mice at 6 months of age (M). Western blotting of the striatum revealed elevated expression of mHTT aggregates and decreased expression of Darpp32, a marker of multi-spiny neurons (MSNs) (Figure 1A) in HD mice, with a significant difference compared to the WT control (Figures 1C,D). Additionally, striatal expression of astrocyte marker (GFAP) and microglia cell (Iba1) significantly increased in HD compared to WT, while the expression of neuron marker (NeuN) remained normal (Supplementary Figures S1A–D). Consistent with the WB results, there was a clear mHTT aggregation and reduction of Darpp32 positive cells in striatum sections of HD mice (Figures 1B,E,F). Similarly, GFAP+ and Iba1+ cells were increased in the striatum of HD mice compared to the WT, while number of NeuN+ cells wasn’t reduced (Supplementary Figures S1E–H). Behaviorally, the HD mice exhibited a shorter latency to fall from a rotating rod (Figures 1G,H) and took longer to cross a balance beam (Figures 1I,J) than WT mice. Therefore, at 6 M of age, R6/1 mice displayed severe HD pathology in the striatum and motor dysfunction.

Having confirmed the HD-related phenotype in the brain and motor function, we performed visual behavioral tests on 6-month-old R6/1 HD model mice and their litter-mate WT controls to evaluate their visual function.

In the dark–light transition test, despite a slight trend towards reduced time spent in the black box among HD mice, no statistically significant difference was observed compared to the WT controls (Figures 1K,L). However, the optomotor system test revealed a significant decline in visual acuity in HD mice compared to their WT counterparts (Figures 1M,N). These findings indicate that HD not only impairs motor function but also affects visual acuity, potentially due to HD-induced retinal cell degeneration and dysfunction.

### 3.2 Reduced retinal light responses in R6/1 mice with age

**Figure 2:**
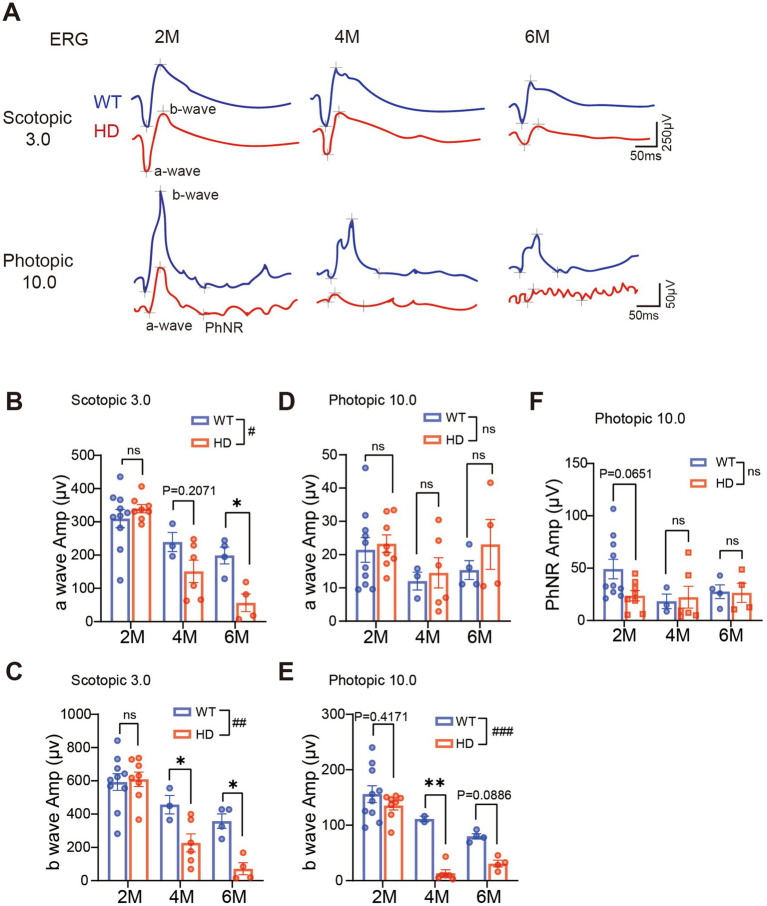
Reduced retinal light responses in R6/1 mice with age. **(A)** Example of ERG traces to flashes at 3.0 cd.s/m^2^ under dark adaptation (scotopic) or 10.0 cd.s/m^2^ under light adaptation (photopic) from two groups with R6/1 mouse (red) and WT mouse as control (blue) at 2 M, 4 M, and 6 M. **(B,C)** Average peak amplitudes of a-wave **(B)** and b-wave **(C)** at scotopic 3.0 cd.s/m^2^. **(D,E)** Average peak amplitudes of a-wave **(D)** and b-wave **(E)** at photopic 10.0 cd s/m^2^. **(F)** Average peak amplitudes of PhNR at photopic 10.0 cd s/m^2^. Data are expressed as mean ± SEM; #, *p* < 0.05, ##, *p* < 0.01, ###, *p* < 0.001, two-way ANOVA analysis as groups; *p < 0.05, **p < 0.01, ***p < 0.001, multiple comparisons by two-way ANOVA. Ns, not significantly different (*p* > 0.5). WT, C57BL/6 J, HD, Huntington’s disease. Each dot in the graph represents the data from one animal.

As the HD symptom gradually develops in R6/1 mice with age, we evaluated their visual functions at the age of 2 M (pre-symptomatic stage) to 6 M (severe symptomatic stage). The retinal light responses under dark adaptation and light adaptation (Figure 2A) were recorded by ERG recording.

Under dark adaptation (scotopic condition), rods are the primary photoreceptors that respond. At 2 M of age, the amplitude of scotopic ERG a-wave (representing the group light responses of photoreceptors) was comparable between HD and WT. However, a decline in a-wave amplitude was observed in HD mice starting from 4 M (Figures 2A,B). Similarly, a significant reduction in the b-wave amplitude (representing the light response of ON bipolar cells) was also observed in HD mice from 4 M onward (Figure 2C). The reduction was less pronounced when the animals were exposed to light intensities below 3.0 cd.s/m^2^ (Supplementary Figures S2A,C–F).

After light adaptation (photopic condition) that bleaches the rod’s responses, mainly cones respond. Across all ages tested, the photopic a-wave amplitude remained similar to that of WT (Figure 2D). However, the photopic b-wave amplitude decreased in HD starting from 4 M (Figure 2E). The PhNR which represents the light responses of retinal ganglion cells (RGCs) remained comparable to that of WT mice at all ages tested (Figure 2F). A similar decay in ERG responses was also observed when animals were stimulated with weaker light flashes (Supplementary Figures S2B,G,H). In summary, our recording demonstrated a gradual decline in both rod and cone responses in HD starting from 4 M of age.

### 3.3 Impaired photoreceptor structure in R6/1 retina since 2 M

Abnormal photoreceptors have been documented in R6/1 mice as early as 7 weeks of age (Batcha et al., 2012), yet the progression of photoreceptor degeneration over time remains unclear. To investigate the morphological abnormalities in the retinas of HD mice at different ages, we conducted DAPI staining on the retinas of R6/1 mice at 2, 4, and 6 M of age. Notably, from 4 M onwards, the retinas of R6/1 mice exhibited a distinct wavy pattern near the optic disc, particularly prominent in the outer nuclei layer (ONL) where photoreceptor somas are situated. In contrast, the retinas of WT control mice remained flat without any such waves (Supplementary Figure S3A).

**Figure 3:**
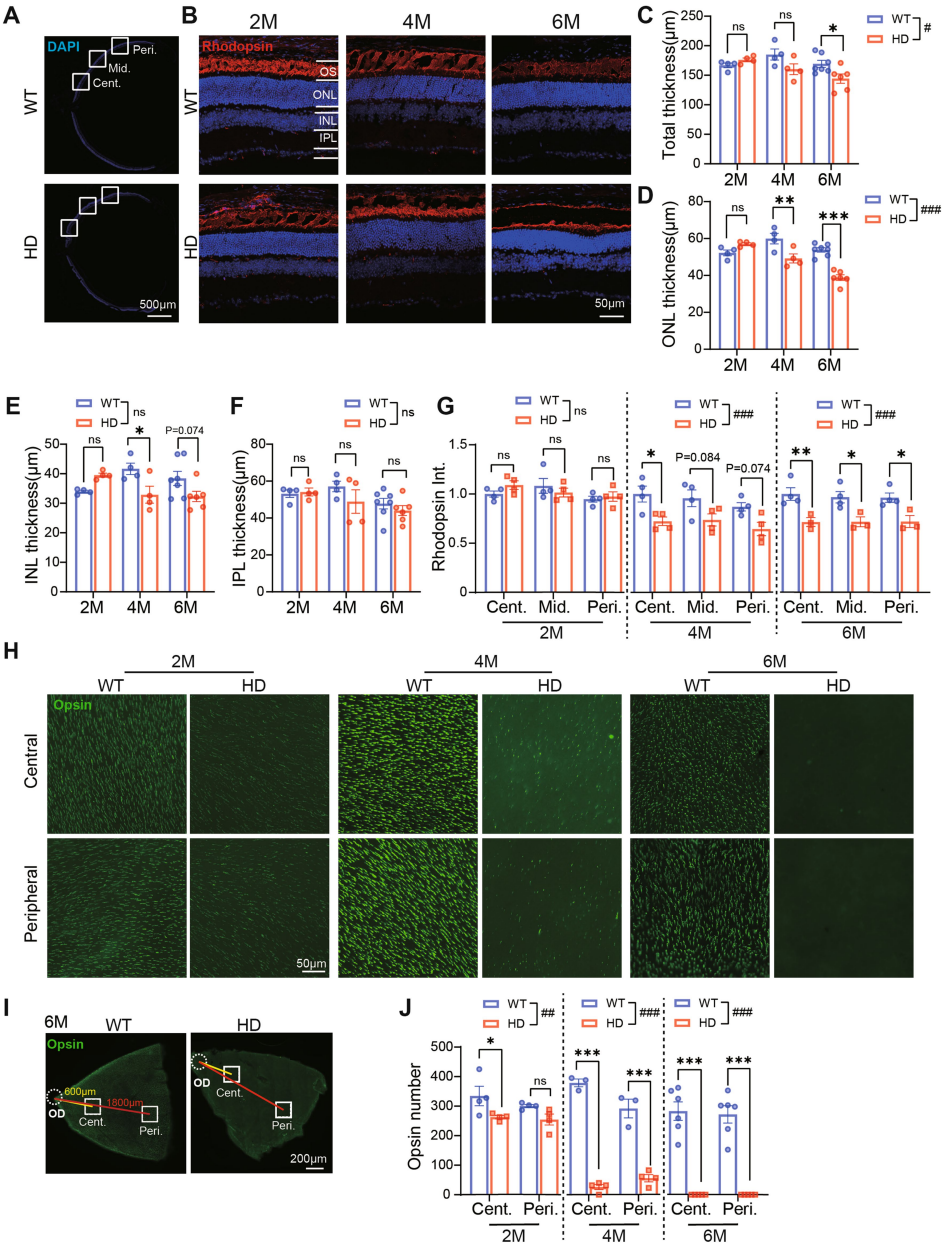
Impaired photoreceptor structure in R6/1 retina since 2 M. **(A)** Images of DAPI staining of retina sections with boxed regions showing the center, middle, and peripheral regions away from the optic disk center. **(B)** Images of retinal sections stained with Rhodopsin (red, marker of rod photoreceptor outer segment) and DAPI (blue) from both animal groups at 2 M, 4 M, and 6 M. **(C–F)**The thickness of total retinal layers **(C)**, the ONL **(D)**, the INL **(E)**, and the IPL **(F)** in WT and HD at the center region (500 μm away from the optic disk center) across ages. **(G)** The mean fluorescent intensity of rhodopsin in retinal sections at 2 M, 4 M, and 6 M. In both groups, retinal sections were evaluated from the center to the peripheral regions. **(H)** Staining of Red/Green opsin in the center and peripheral regions from whole-mount retina of WT and HD mic across ages. **(I)** Illustrations of the center and peripheral regions on the whole-mount retina. **(J)** Number of cone OS stained by Opsin per image (diameter: 200 μm). Data are expressed as mean ± SEM; #, *p* < 0.05, ##, *p* < 0.01, ###, *p* < 0.001, two-way ANOVA analysis as groups; *p < 0.05, **p < 0.01, ***p < 0.001, multiple comparisons by two-way ANOVA. Ns, not significantly different (*p* > 0.5). WT, C57BL/6 J, HD, Huntington’s disease; OD, optic disc; OS, outer segment; ONL, outer nuclei layer; INL, inner nuclei layer; IPL, inner plexiform layer; Cent., center; Mid, middle; Peri., peripheral. Each dot in the graph represents the data from one animal.

Subsequently, we measured the thickness of the retina from center to peripheral regions (Figure 3A). At 2 M and 4 M, the total retinal thickness of HD mice was similar to the control. However, at 6 M, the total retinal thickness in HD mice was significantly thinner than that of controls (Figure 3C). Further analysis revealed that the ONL (Figure 3D) and the inner nuclear layer (INL, containing bipolar cells, horizontal cells, and amacrine cells, Figure 3E) were thinner in HD mice starting from 4 M, while the inner plexiform layer (IPL, where synaptic connections among bipolar, ganglion, amacrine, and horizontal cells occur) remained unaffected (Figure 3F). The reduction in ONL thickness was more severe at 6 M compared to 4 M, suggesting that mHTT significantly impacts the photoreceptor cell layer. The similar results about retinal thickness were also shown in the middle and peripheral regions (Supplementary Figures S3C,D).

To further elucidate the structure of cone and rod photoreceptor cells, we conducted immunofluorescence staining for rhodopsin (a marker of rod outer segments, OS) on retinal sections (Figure 3B) and opsin (a marker of cone OS) on whole-mount retina (Figure 3H). In WT mice, a densely packed cone OS and a heavily stained rod OS layer were consistently observed across all ages. Conversely, in HD mice, there was a gradual age-related loss of both cone OS and rod OS. Quantifying the fluorescent intensity of rhodopsin staining in the OS layer revealed a significantly weaker signal in HD mice compared to WT, starting from 4 M of age (Figure 3G), particularly in the central regions. Notably, the loss of cone OS was more severe than that of rods, as evidenced in the whole-mount retina staining (Figure 3H). Specifically, in HD mice, a significant reduction in opsin number was observed in the central regions as early as 2 M, and by 6 M, there was a complete loss of opsin staining in both the central and peripheral regions (Figures 3I,J). These findings indicate that retinal photoreceptors, including both cone and rod photoreceptors, are impaired in HD mice. The destruction of cone photoreceptors initiates as early as 2 M, while the destruction of rod photoreceptors occurs later.

### 3.4 Abnormal bipolar cell dendrites and synaptic connections in the outer HD retina at 6 M

In a normal retina, bipolar cells establish synaptic connections with photoreceptors in the outer plexiform layer (OPL) and with ganglion cells in the inner plexiform layer (IPL) through their dendrites and axons, respectively. Given the impairment of photoreceptors in HD retina, we investigated whether this might also affect bipolar cells and their synaptic connections in HD mice.

**Figure 4:**
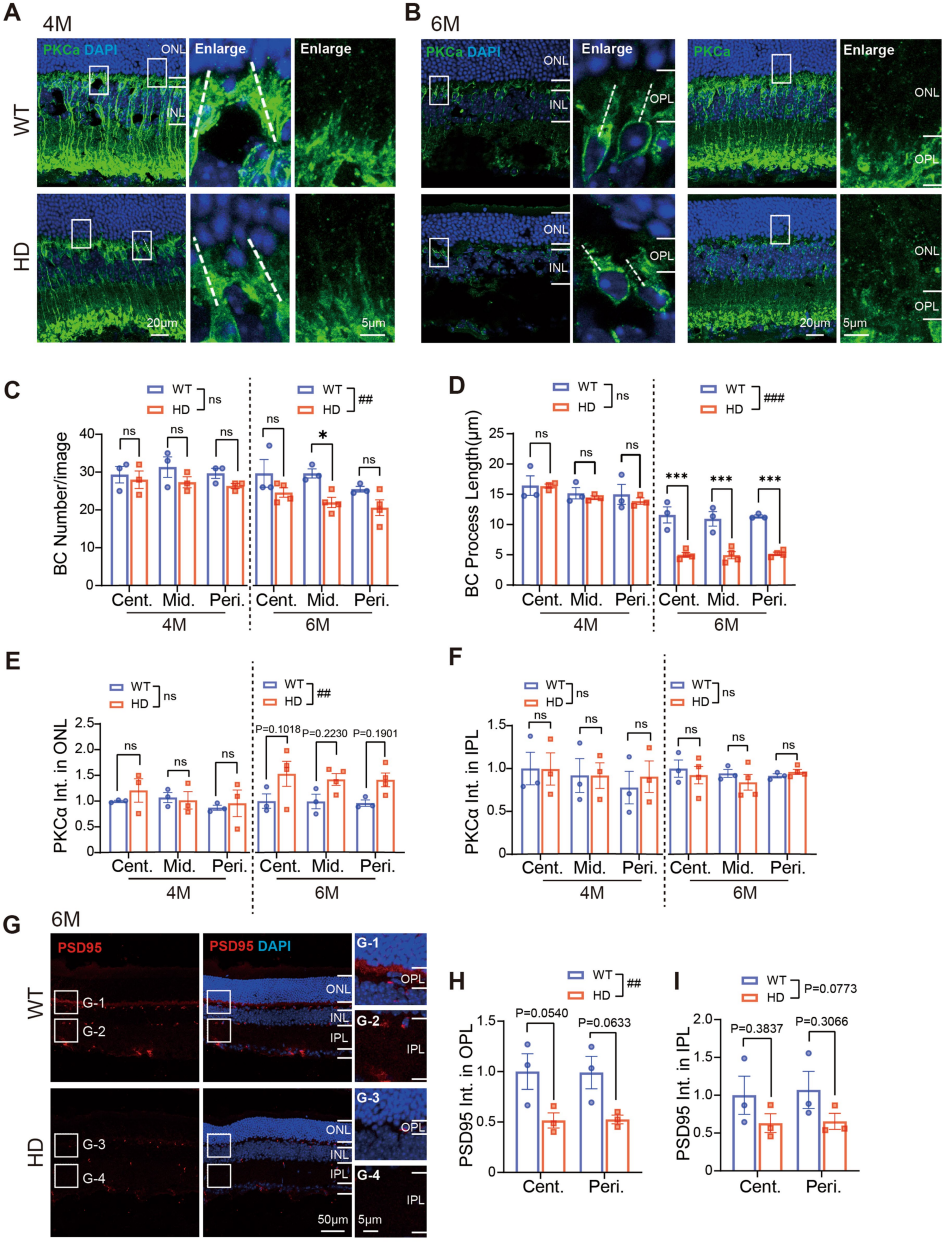
Abnormal bipolar cells and synaptic connections in outer HD retina at 6 M. **(A,B)** Images of retinal sections stained with PKCα (green, marker of rod bipolar cell) at 4 M **(A)** and 6 M **(B)**. Boxed regions were enlarged and shown on the right panels. A white dotted line marked the length of the major dendrite of the cell. **(C)** Number of bipolar cells (BC) per image with a size of 160 × 160 μm from central to peripheral regions at 4 M and 6 M. **(D)** Length of BC dendrites in each image from central to peripheral regions. **(E,F)** Mean fluorescent intensity of PKCα staining in retinal ONL **(E)** or IPL **(F)** regions. **(G)** Images of retinal sections stained with PSD95 (red) at 6 M. Inserts illustrate magnified images from the boxed regions. **(H,I)** The mean fluorescent intensity of PSD95 staining in retinal OPL **(H)** or IPL **(I)** at 6 M. Data are expressed as mean ± SEM; ##, *p* < 0.01, ###, *p* < 0.001, two-way ANOVA analysis as groups; *p < 0.05, **p < 0.01, ***p < 0.001, multiple comparison by two-way ANOVA. Ns, not significantly different (*p* > 0.5). WT, C57BL/6 J, HD, Huntington’s disease; ONL, outer nuclei layer; OPL, outer plexiform layer, INL, inner nuclei layer; IPL, inner plexiform layer; BC, bipolar cell; Cent., center; Mid, middle; Peri., peripheral. Each dot in the graph represents the data from one animal.

We began by examining rod bipolar cells, the primary type of bipolar cells, using immunofluorescence staining with PKCα. Since rods remained relatively normal in HD retina until 4 M, we initiated our examination of rod bipolar cells at this time point (Figures 4A,B). At 4 M, both the number of rod bipolar cells and the length of their major dendritic processes (illustrated by dotted lines in Figures 4A,B) were normal in HD. However, by 6 M, we observed a reduction in the number of rod bipolar cells compared to WT mice (Figure 4C), accompanied by a significant shortening of dendritic length in all regions (Figure 4D). Additionally, while the dendritic processes of most bipolar cells became shorter in HD mice, abnormal expression of PKCα was also noted in the ONL, suggesting an abnormal outsprouting of rod bipolar cell processes, as reported by Batcha (Batcha et al., 2012). Quantification of fluorescent intensity in the ONL confirmed a stronger expression in HD mice compared to WT at 6 M (Figure 4E). In contrast, the expression of PKCα in the IPL, where rod bipolar cells form synapses with amacrine cells, remained similar to that of WT mice at both 4 M and 6 M (Figure 4F). These findings indicate that HD causes damage to rod bipolar cells at their dendrites by 6 M.

Next, we examined the synaptic connections in the OPL and IPL by immunostaining for PSD95, a crucial postsynaptic protein essential for maintaining synaptic structure and function (Figure 4G). In HD mice, we observed a significant decrease in the mean fluorescence intensity of PSD95 in the OPL (Figure 4H) and a tendency towards decline in the IPL at 6 M (Figure 4I). These results further confirm the presence of abnormal synaptic connections in the outer retina of HD mice.

### 3.5 Reduced number of RGCs and abnormal optic nerve fiber in HD mice at 6 M

**Figure 5:**
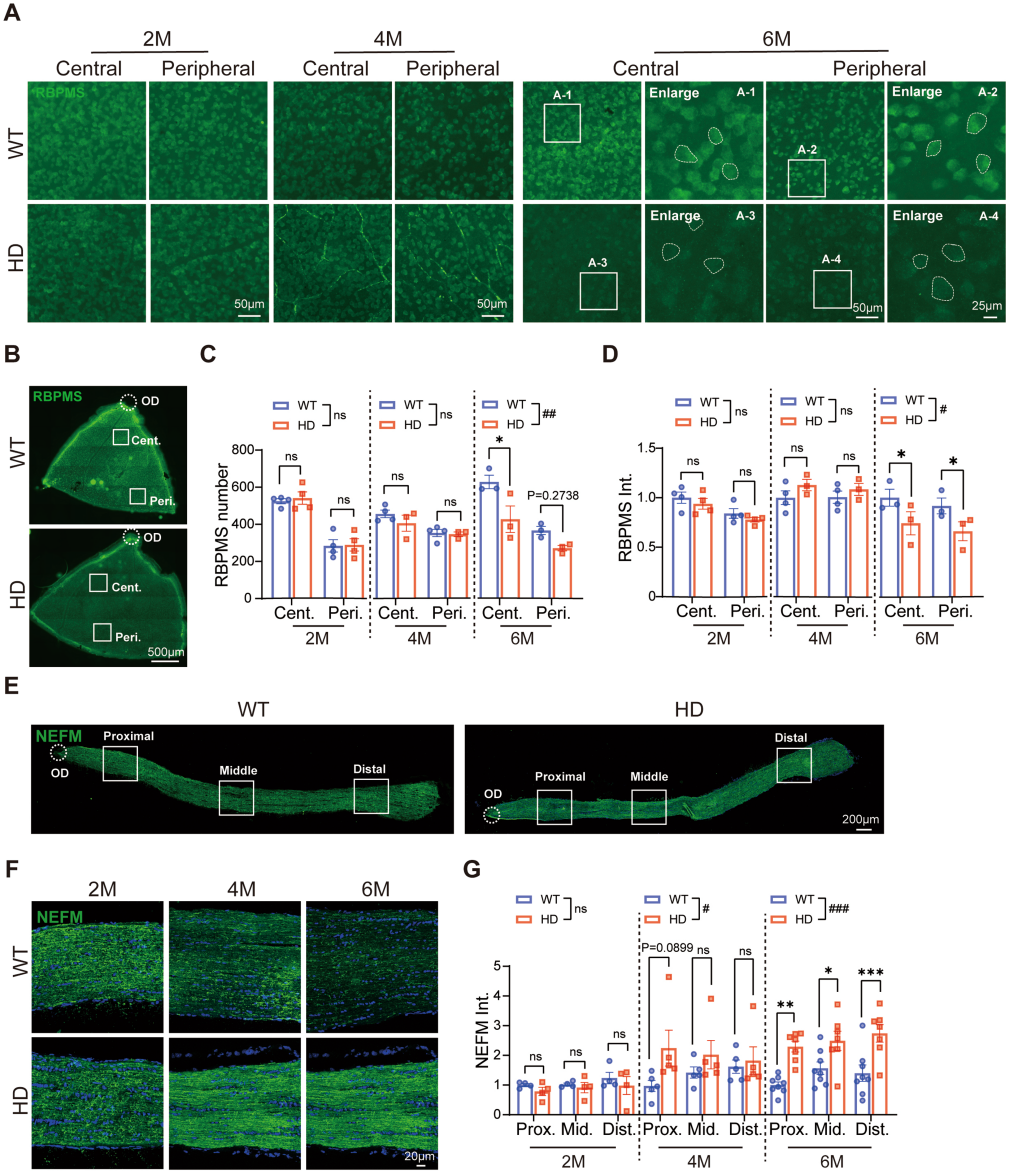
Reduced number of ganglion cells and abnormal optic nerve fibers in HD mice at 6 M. **(A)** Images of RBPMS (green, a marker of RGCs) staining in retinal whole-mounts at central and peripheral regions in different animal groups at 2 M, 4 M, and 6 M. The boxed regions from the 6 M retina were enlarged and shown in A1-A4, illustrating the RBPMS staining in RGCs soma (circled by dotted lines). **(B)** Illustration of center and peripheral regions in the whole-mount retina stained by RBPMS. **(C,D)** The mean number of RGCs **(C)** and fluorescent intensity of RBPMS **(D)** in retinal whole-mount from the central to the peripheral regions across ages. **(E)** Confocal images of NEFM (green) staining in optic nerve sections from proximal to distal regions at 2 M. **(F)** Confocal images of NEFM (green) staining in the optic nerve at proximal region across ages. **(G)** The mean fluorescent intensity of NEFM in optic nerve sections across regions and ages. Data are expressed as mean ± SEM; #, *p* < 0.05, ##, *p* < 0.01, ###, *p* < 0.001, two-way ANOVA analysis as groups; *p < 0.05, multiple comparison by two-way ANOVA. Ns, not significantly different (*p* > 0.5). WT, C57BL/6 J, HD, Huntington’s disease; OD, optic disc; RGC; retinal ganglion cell; Cent., center; Mid, middle; Peri., peripheral; Prox., proximal; Dist., distal. Each dot in the graph represents the data from one animal.

RGCs, found in the retina’s final order, transmit visual signals to the brain. To investigate whether RGCs in R6/1 mice showed abnormal characteristics, we conducted immunofluorescence staining of RBPMS (an RGC marker) on whole-mount retinas across various ages (Figure 5A). We quantified the number of ganglion cells in both the center and periphery of the retina (Figure 5B), as well as their fluorescence intensity. At 2 and 4 M, the density of RGCs was similar between HD and WT mice in both regions. However, at 6 M, the number of RGCs significantly decreased in the central region of HD mice (Figure 5C). Additionally, the staining of RBPMS in RGC somas weakened in HD mice at 6 M, displaying granularity and blurred cytosolic contours compared to the clear edges in WT mice (Figure 5A, enlarged regions). The average fluorescence intensity of RBPMS was also significantly lower in both the center and periphery of HD mice at 6 M (Figure 5D). These data indicated an abnormality in the inner retina of HD mice.

We further analyzed the structure of the optic nerve, composed of RGC axons projecting to brain regions. Immunofluorescence staining of neurofilament proteins with NEFM was used to assess the optic nerve structure from the proximal (eyeball connection) to the distal end (brain connection) (Figure 5E). The results showed a significant increase in fluorescence intensity in HD mice, spanning from the central to peripheral regions of the optic nerve at 6 M (Figures 5E–G). This indicated an abnormal optic nerve structure in HD mice. Thus, R6/1 mice exhibited notable abnormalities in both the soma and axons of RGCs. These abnormalities in both the outer and inner retina may have contributed to the reduced visual function observed in HD mice.

### 3.6 Inflammatory reactions happened in the HD retina since 4 M

In both KI mouse and monkey models of HD, reactive gliosis—marked by astrocyte and microglia activation—is recognized as an early brain pathology (Yu et al., 2003; Palfi et al., 2007). Similarly, the reactive gliosis has been observed in R6/1 at 7 weeks of age (Batcha et al., 2012). Therefore, we examined how gliosis progresses in the R6/1 retina with age.

**Figure 6:**
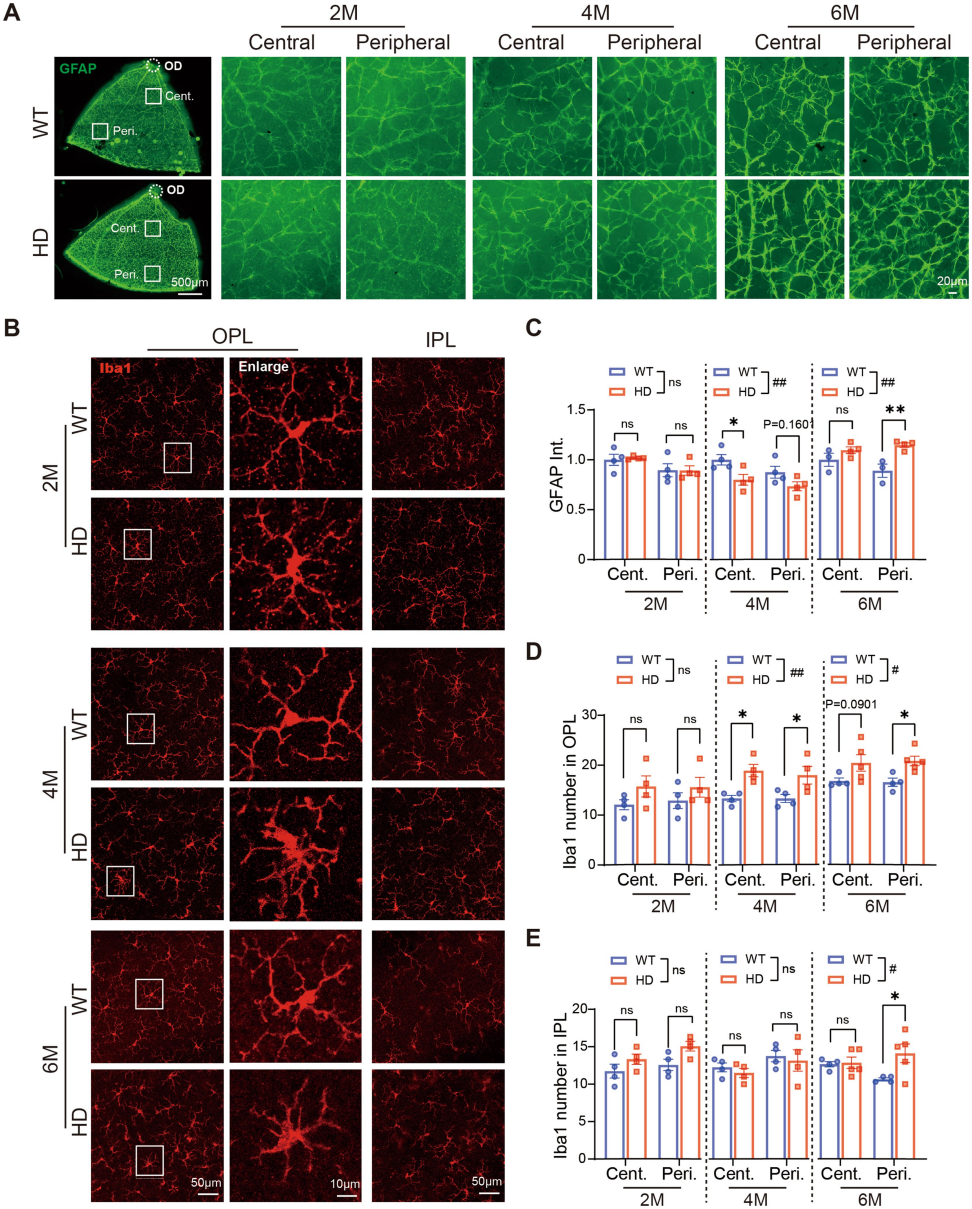
Inflammatory reactions happen in the HD retina at 4 M. **(A)** Images of GFAP (green) staining in retinal whole-mounts at 6 M, with central and peripheral regions shown on the right across ages. **(B)** Images of Iba1 (red) staining in the center region of retinal whole-mount at the OPL and IPL, with boxed regions in OPL enlarged and shown on its right at 2 M, 4 M, and 6 M. **(C)** The mean fluorescent intensity of GFAP per image (320 × 320 μm) in retinal whole-mounts from central to peripheral regions across ages. **(D,E)** The number of Iba1+ cells in OPL **(D)** or IPL **(E)** at central and peripheral regions of retinal whole-mount across ages. Data are expressed as mean ± SEM;#, *p* < 0.05, ##, *p* < 0.01, two-way ANOVA analysis as groups; *p < 0.05, **p < 0.01 multiple comparisons by two-way ANOVA. ns, not significantly different (*p* > 0.5). WT, C57BL/6 J, HD, Huntington’s disease; OD, optic disc; OPL, outer plexiform layer; IPL, inner plexiform layer; Cent., center; Peri., peripheral; Int., intensity mean value. Each dot in the graph represents the data from one animal

First, we used immunostaining with GFAP antibody to observe astrocyte changes on whole-mount retinas (Figure 6A). GFAP expression was normal in HD mice at 2 M, but astrocyte branches became sparse at 4 M and much denser at 6 M compared to WT mice. Measuring GFAP fluorescent intensity revealed a significant decrease in the central retina at 4 M, followed by a significant increase in both central and peripheral regions at 6 M (Figure 6C). At 6 M, astrocytes in HD mouse retinas showed typical activated features, including increased cytosolic size and shortened axons, which are key inflammatory response markers (Figure 6C).

Next, we assessed microglia status in HD retinas by immunostaining with Iba1 (Figure 6B). At 2 M, microglia morphology and density were similar in HD and WT mice. However, from 4 M onwards, microglia in the OPL of R6/1 retinas exhibited amoeboid morphology with swollen somas and shrinking dendrites, indicative of microglia activation (Figure 6C, enlarged region). This morphological change was less pronounced in the IPL of HD retinas compared to the OPL. Counting Iba1+ cells showed a significant increase in microglia number in the OPL since 4 M, particularly in the peripheral region (Figure 6D), and to a lesser extent in the IPL (Figure 6E).

The activation of both astrocytes and microglia in HD mouse retinas suggests that an inflammatory response occurs as early as 4 M, potentially a significant contributor to HD retinopathy.

### 3.7 mHTT aggregates in HD retina and optic nerve since 2 M

**Figure 7:**
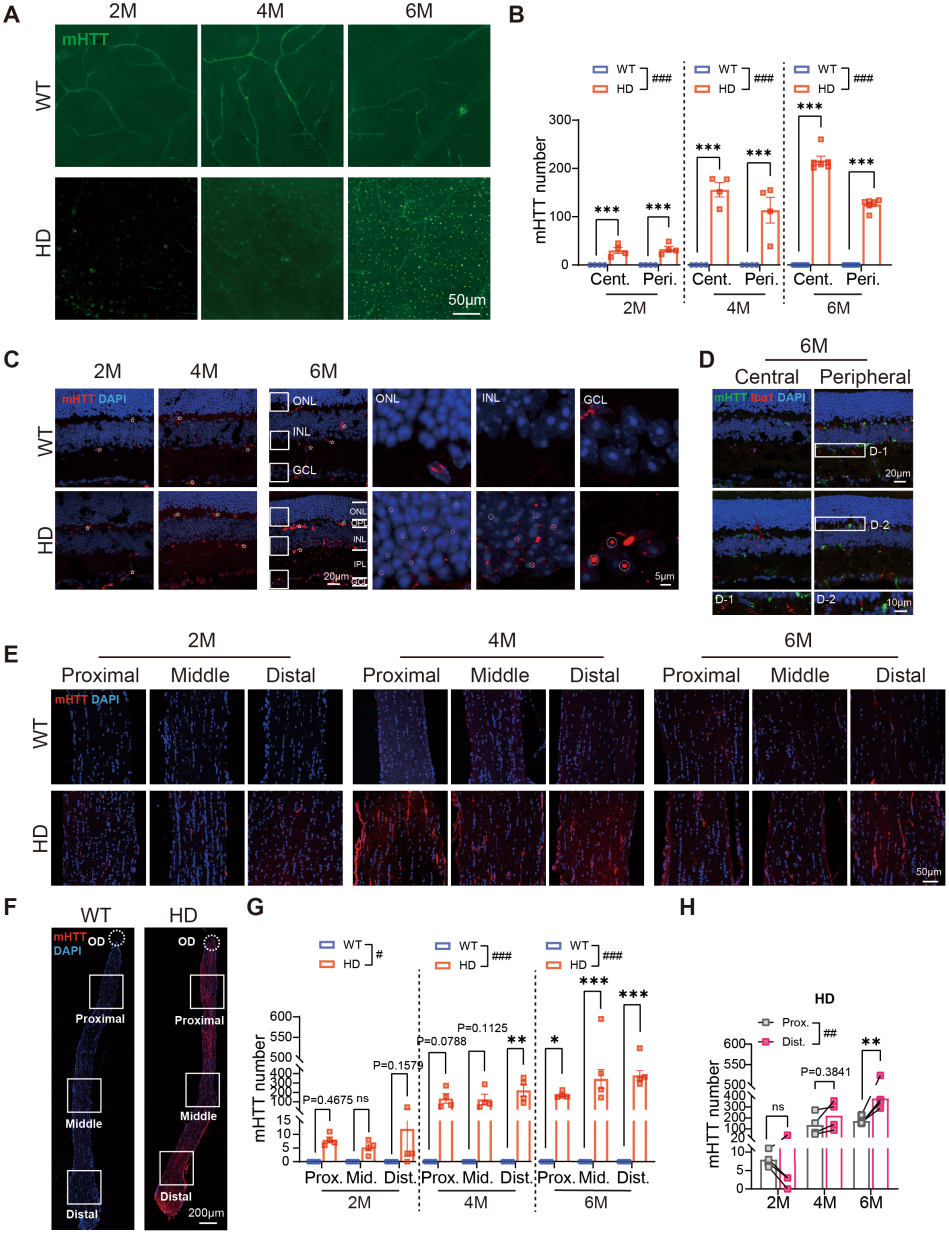
mHTT aggregates in all layers of the HD retina and the optic nerve. **(A)** Staining of mHTT (green) in the center region of retinal whole mounts at 2 M, 4 M, and 6 M. **(B)** The number of mHTT per image (diameter: 200 μm) in retinal whole-mounts from central to peripheral regions across ages. **(C)** Staining of mHTT (red) in the center regions of retinal sections at 2 M, 4 M, and 6 M. Inserts illustrate magnified images from the boxed regions at ONL, INL, and GCL. White stars: vessel blood. **(D)** Staining of mHTT (green), Iba1 (red, marker of microglia), and DAPI (blue) in retinal sections from central to peripheral at 6 M, with boxed regions enlarged and shown in the bottom. **(E,F)** Confocal images of mHTT (red) and DAPI (blue) staining in optic never sections from both animal groups across regions and ages, with an illustration of the regions shown in F. **(G)** The number of mHTT in optic nerve from proximal to distal regions in two animal groups across ages. (**H**) Comparison of the mHTT number in the proximal and distal regions of optic nerve in HD mice across ages. Data are expressed as mean ± SEM; #, *p* < 0.05, ##, *p* < 0.01, ###, *p* < 0.001, two-way ANOVA analysis as groups; *p < 0.05, **p < 0.01, ***p < 0.001, multiple comparison by two-way ANOVA. Ns, not significantly different (*p* > 0.5). WT, C57BL/6 J, HD, Huntington’s disease; OD, optic disc; ONL, outer nuclei layer; OPL, outer plexiform layer; INL, inner nuclei layer; IPL, inner plexiform layer; GCL, ganglion cell layer; Cent., center; Mid, middle; Peri., peripheral; Int., intensity mean value; Prox., proximal; Mid, middle; Dist., distal. Each dot in the graph represents the data from one animal.

To investigate the potential link between mHTT and retinal abnormalities in HD, we performed immunostaining on whole-mount retinas. In WT controls, the mHTT antibody stained only a few blood vessels. Conversely, in HD retinas, mHTT puncta were visible from 2 M onwards and increased in number with age, distributing across the center to the periphery (Figure 7A, center regions). The density of mHTT was significantly higher in HD mice compared to controls at all ages, with a higher concentration in the center than the periphery (Figure 7B).

A closer examination of mHTT distribution across retinal layers revealed that a few mHTT puncta appeared in the ganglion cell layer (GCL) of R6/1 retinas at 2 M, increasing in number in the GCL, IPL, and OPL at 4 M. By 6 M, numerous mHTT puncta were present in the deep layers of the INL (Figures 7C,D). The size of mHTT puncta increased from the outer retina to the inner retina, with the smallest puncta in the ONL, larger puncta in the INL, and the largest in the GCL at 6 M (Figure 7C, circled dots). In contrast, the WT retina primarily showed staining of blood vessels (white stars in Figure 7C). Comparison of mHTT levels in different regions showed higher values in the central compared to the peripheral region at 6 M.

We further examined the distribution of mHTT in the optic nerve. As early as 2 M, mHTT puncta appeared in the DAPI-stained nuclei in the optic nerve, accompanied by staining of some blood vessels. In contrast, the optic nerve of WT animals showed virtually no mHTT puncta (Figure 7E). Notably, the number of mHTT puncta in the optic nerve of R6/1 mice was significantly higher than in WT mice, spanning from the proximal to the distal end (Figures 7F,G). In R6/1 mice, a bright fluorescent signal was observed at the distal end of the optic nerve, with only a faint signal present at the proximal end. Comparison of mHTT levels between the proximal and distal ends of the optic nerve further confirmed the higher expression of mHTT at the distal end (Figure 7H).

In summary, our findings revealed that mHTT aggregates in all retinal layers of HD mice, with the degree of aggregation influenced by cell type and centrifugal location. Additionally, mHTT aggregates were also observed in the optic nerve. This aggregation may play a significant role in the observed abnormalities in retinal structure.

## 4 Discussion

In our current study, we investigated whether the R6/1 transgenic mouse model of HD exhibited abnormalities in both the outer and inner retina, beyond the striatum, and how retinopathy progressed with age. At 6 M, R6/1 mice displayed severe HD symptoms in the striatum and impaired visual acuity. Retinal light responses gradually declined starting from 4 M. Structurally, there was a pre-symptomatic loss of cone opsin, followed by mild rhodopsin loss, shortened rod bipolar dendrites, and inflammation at the symptomatic stage. Additionally, mild impairment of ganglion cells in the inner retina was observed at 6 M. Our findings revealed that retinopathy affects both the outer and inner retina, with earlier progression in the outer retina.

Abnormalities in the outer retina have been reported in HD patients and animal models. However, reports on outer retinopathy in HD patients are inconsistent. Some studies have shown increased ERG responses in HD patients (Pearl et al., 2017), while others have reported decreased responses (Knapp et al., 2018). Similarly, color vision was reported to be reduced in some HD patients (Gulmez Sevim et al., 2019) but remained normal in others (Dusek et al., 2023). Therefore, further evidence is needed to determine whether outer retinopathy can serve as a reliable indicator of HD in clinical settings. In contrast, all animal models have consistently shown outer retinopathy. In R6/1 and R6/2 mice, loss of cone opsin but not cones was reported, along with a wavy retina and outsprouting of bipolar processes into the ONL. Consistent with the loss of cone opsin, severe reduction in the cone light responses measured by ERG recording was reported in both R6/1 and R6/2 mice (Helmlinger et al., 2002; Batcha et al., 2012; Cano-Cano et al., 2024; Ragauskas et al., 2014). A recent study further revealed the severely damaged membrane disks in R6/2 photoreceptors at the ultrastructural level at the symptomatic stage (Mazur-Michałek et al., 2022). Our current study confirmed the gradual loss of cone opsins and cone responses with age and further reported the reduction of rhodopsin from rods at a later stage (since 4 M) than the loss of cone opsin (since 2 M). The reduced scotopic ERG at dim flash supported the loss of rod function in R6/1 mice (Supplementary Figure S2). The consistent results on the outer retinopathy in HD mouse models support the use of outer retinal function (assessed by ERG) as a non-invasive method to monitor HD progression and evaluate treatment effectiveness.

On the other hand, in most clinical studies, the major pathology observed in HD patients was in the inner retina, especially the thinning of the RNFL layer, which has therefore, been proposed as a biomarker for HD disease progression. However, a recent study by Dusek et al. (2023) found minimal changes in global RNFL thickness and macular area in HD patients, rendering them unsuitable as biomarkers. Similarly, inconsistent observations of inner retinopathy have been reported in animal models. In R6/2 mice, despite age-dependent accumulation of mHTT in RGCs and the optic nerve, no degeneration of RGCs or the optic nerve was observed, even at later stages (Ragauskas et al., 2014). However, a recent study revealed thinning of myelin sheaths in the optic nerve of R6/2 mice at the symptomatic stage, suggesting potential impairment of RGCs (Mazur-Michałek et al., 2022).In our current study on R6/1 mice, despite a normal PhNR response as WT, we observed the reduced RGCs density and their expression of RBPMS at the symptomatic stage (6 M). These findings indicate the occurrence of inner retinopathy. Additionally, the abnormal rise of neurofilament protein expression in R6/1 optic nerve suggested structural change in the optic nerve. The activation of astrocytes who localize in the same layer as RGCs, further supports the occurrence of late-stage inner retinopathy. Therefore, our study using R6/1 also endorses the idea of using the late-stage inner retina structural change as a tool to evaluate the therapeutic intervention on HD symptoms in animal models. Further studies by *in vivo* monitoring of the RNFL changes in R6/1 mice with age may help validate this idea.

The accumulation of mHTT may contribute to retinopathy in R6/1 mice at a late stage, but may have limited impact at early stages. At 6 M, we observed a higher accumulation of mHTT in the central regions of the retina than in the peripheral. This was accompanied by a greater loss of RGCs, thinning of the ONL, and out-sprouting of rod bipolar cells in the central region compared to the peripheral region. The correlation of the extent of retinopathy with mHTT accumulation at different centrifugal distances, therefore, supports the cellular toxicity induced by mHTT aggregation, as observed in the brain (Arrasate and Finkbeiner, 2012) at the late stage of HD.

However, the retinopathy in the outer retina appeared as early as 2 M, even when mHTT aggregation is primarily confined to the inner retina. This indicates that the early outer retinopathy may not be directly attributed to the mHTT aggregation, but rather due to interference with cellular development by mHTT, as suggested in the cortex of mice and humans (Barnat et al., 2020). Specifically, the mHTT may disrupt the intracellular transport between the outer segment of photoreceptors and their soma, leading to the loss of opsin. While mHTT aggregation appeared in the ganglion cell layer as early as 2 M, the RGCs did not show any abnormalities until 6 M. This indicates that the impact of mHTT aggregation may be cell-type dependent. Indeed, studies have shown that mHTT aggregates in all cell types of the retina except Muller cell (Yefimova, 2023), astrocyte (Ragauskas et al., 2014), or microglia (Figure 7D). The reasons for the differential impact of mHTT and its aggregation on different cell types require further investigation.

In the retina, activation of glial cells indicates inflammation. In our study, we observed the activation of microglia cells in the outer R6/1 retina at 4 M, followed by activation in the inner retina at 6 M. Similarly, astrocytes were activated at 6 M. The temporal sequence of glial cell activation coincided with abnormalities in bipolar cells and RGCs but lagged behind the appearance of mHTT aggregation. This suggests that detecting mHTT aggregation may be more pivotal than inflammation for early diagnosis of HD. However, in clinical practice, inflammation detection is preferred due to its higher feasibility, despite the theoretical relevance of protein aggregation detection. Routine blood tests may lack sensitivity for early-stage patients due to low inflammation levels. Thus, exploring alternative sample collection methods, such as intraocular fluid testing, may be a promising direction.

Given that RGCs are connected to the brain via the optic nerve, it is intriguing to consider whether mHTT could be transported from the brain to the retina. The higher accumulation of mHTT in the central retina (connected to the optic nerve) compared to the peripheral region supports this hypothesis. Additionally, higher mHTT aggregation at the distal end of the optic nerve (connected to the brain) compared to the proximal end (connected to the eyeball) further strengthens this idea. However, if mHTT were transported from the brain to the retina, we would expect an earlier expression of mHTT in the optic nerve than in RGCs. Nevertheless, mHTT was observed in both RGCs and the optic nerve as early as 2 M. To clarify this, examining mHTT expression at a time point earlier than 2 M may be necessary.

Using R6/1 to mimic HD patients has certain limitations: although the progression of HD in R6/1 mice is slower than in R6/2 mice, it is still faster than in human patients, potentially failing to fully capture the chronic nature of the disease. However, this rapid progression can be advantageous when evaluating treatment effectiveness or exploring HD mechanisms using this mouse model. Indeed, we have tried to use the current mouse model and methods to test two candidate drugs, but none showed a protective effect on either the retina or brain structure. And we are testing more chemicals.

In summary, our study reveals that abnormalities occur in both the inner and outer retina of R6/1 mice, with earlier progression in the outer retina. These abnormalities are accompanied by decreased light responses and strong inflammatory responses, but they do not coincide with the accumulation of mHTT aggregates. Therefore, the R6/1 mouse retina may be a better model for studying HD mechanisms and evaluating treatment strategies, rather than as a diagnostic tool for HD.

## Data Availability

The original contributions presented in the study are included in the article/Supplementary material, further inquiries can be directed to the corresponding authors.
