# Supplementary figures and images for: Abnormal outer and inner retina in a mouse model of Huntington’s disease with age
Source: Front Aging Neurosci. 2024 Oct 28;16:1434551. doi: 10.3389/fnagi.2024.1434551 (PMC11550939; doi:10.3389/fnagi.2024.1434551)

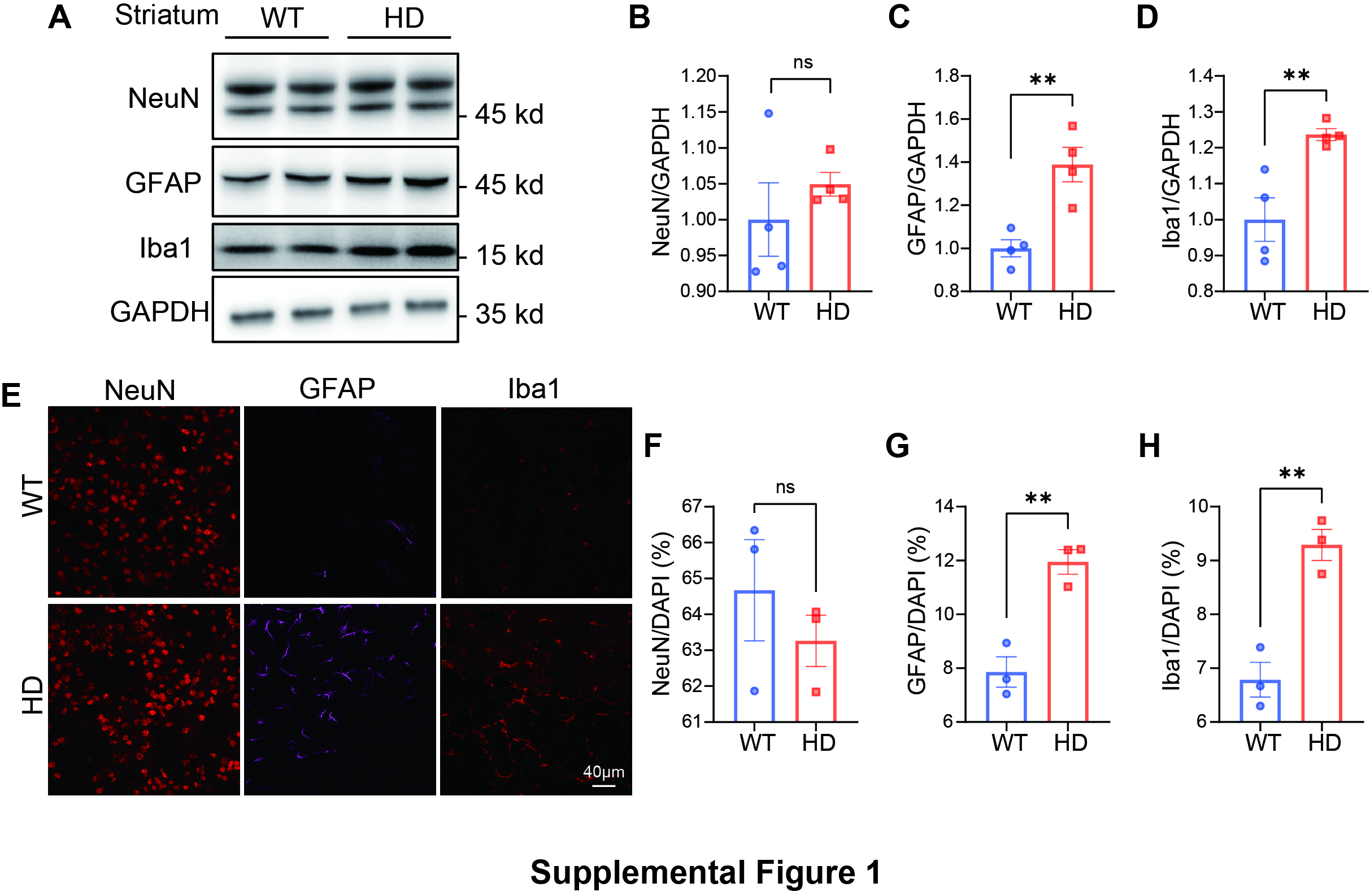

Supplement: Supplementary file 1 [file Image_1.JPEG]

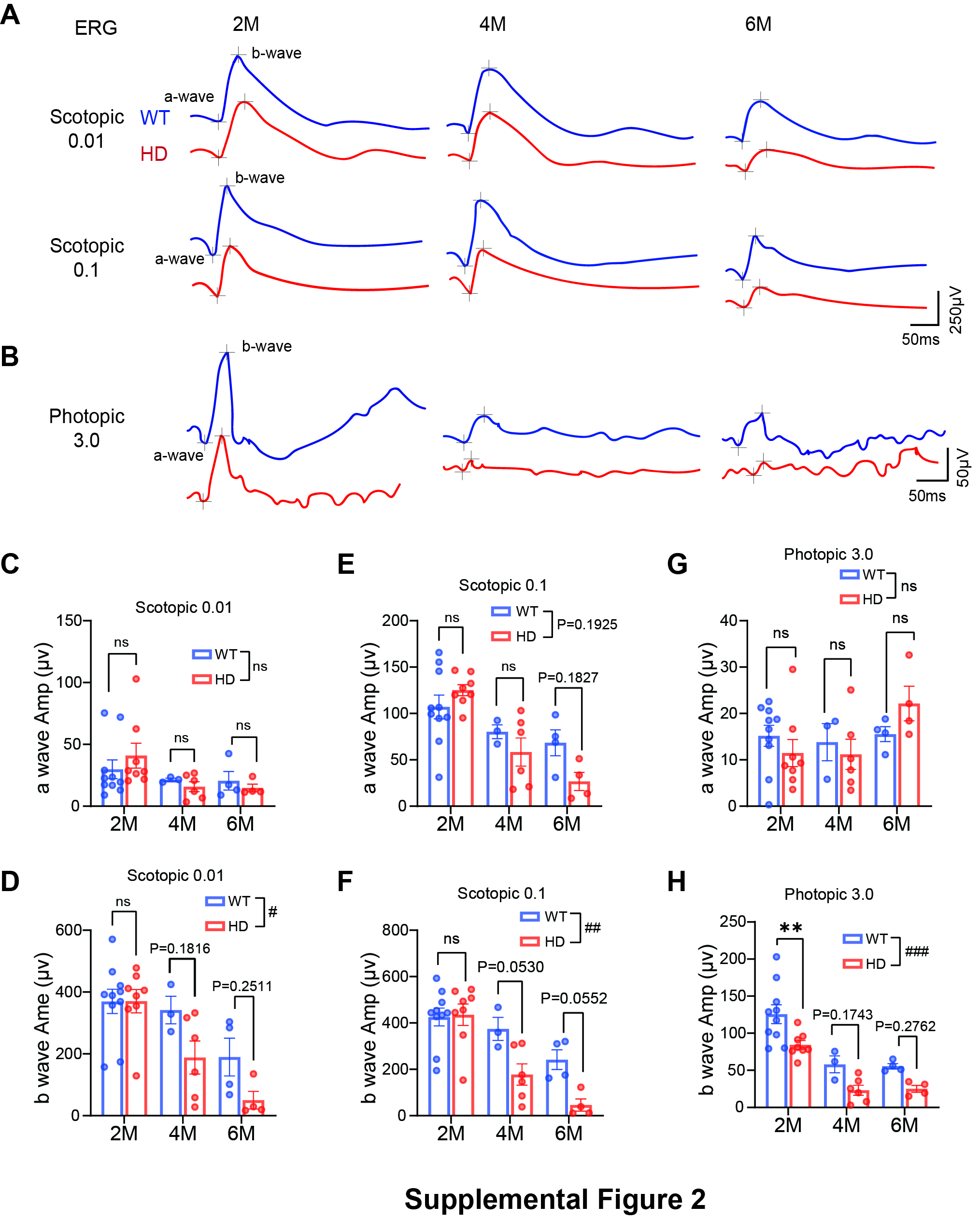

Supplement: Supplementary file 2 [file Image_2.JPEG]

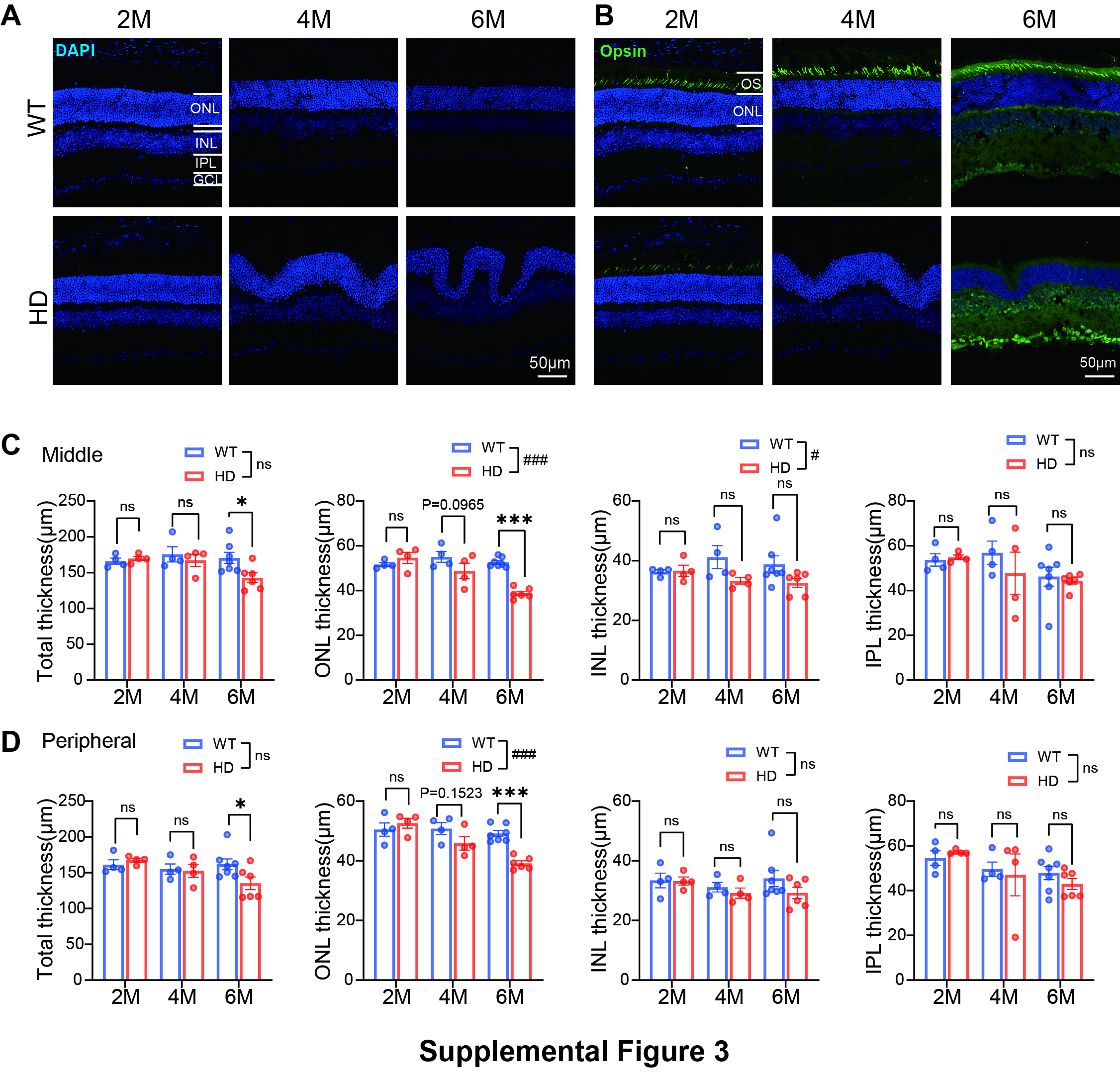

Supplement: Supplementary file 3 [file Image_3.JPEG]
