# Supplementary material for: Abnormal outer and inner retina in a mouse model of Huntington’s disease with age
Source: Front Aging Neurosci. 2024 Oct 28;16:1434551. doi: 10.3389/fnagi.2024.1434551 (PMC11550939; doi:10.3389/fnagi.2024.1434551)
Supplement: Supplementary file 4 [file Table_1.DOCX]

**Supplemental Table 1 The number and gender of mice used in this experiment.**

|  | 2M | | | | 4M | | | | 6M | | | |
| --- | --- | --- | --- | --- | --- | --- | --- | --- | --- | --- | --- | --- |
|  | WT | | HD | | WT | | HD | | WT | | HD | |
|  | F | M | F | M | F | M | F | M | F | M | F | M |
| **Total** | **4** | **6** | **7** | **1** | **9** | **4** | **10** | **7** | **7** | **27** | **8** | **24** |
| Behaviors | 0 | 0 | 0 | 0 | 0 | 0 | 0 | 0 | 0 | 14 | 0 | 14 |
| ERG | 4 | 6 | 7 | 1 | 3 | 0 | 4 | 2 | 0 | 4 | 0 | 4 |
| Western blotting(WB) | 0 | 0 | 0 | 0 | 0 | 0 | 0 | 0 | 0 | 4 | 0 | 4 |
| Immunostaining (IF) | 1 | 4 | 4 | 0 | 6 | 4 | 6 | 5 | 7 | 15 | 8 | 13 |

For HD mice at 6M:


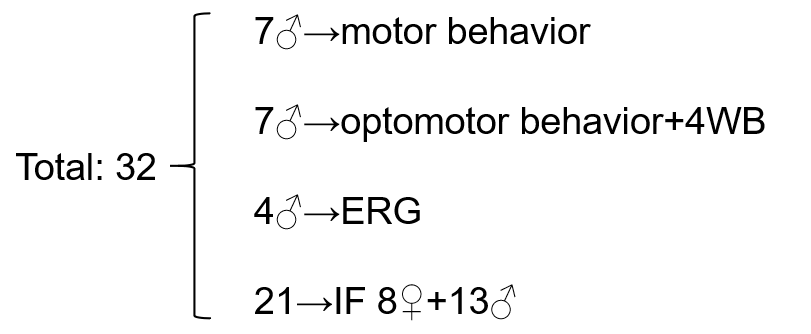


**Supplemental Figure 1. Inflammatory reactions happen in the HD striatum.**

**(A)** Western blotting showing the expression of NeuN (a marker of neurons), GFAP, and Ibal in the striatum of WT and HD. GAPDH served as a loading control. **(B-D)** The ratios of NeuN (B), GFAP (C), or Iba1 (D) expression to GAPDH **(E)** Immunofluorescent staining with anti-NeuN (red) to label neuronal cells, anti-GFAP (purple) to label astrocytes, and Iba1 (red) to label microglia o the striatum slices. **(F-H)** The ratio of NeuN-positive (F), GFAP-positive (G), or Iba1-positive (H) cells of the total cells per image (size of 318 x 318 μm) in mouse striatum. Data are expressed as mean ± SEM; *P < 0.05, **P < 0.01, ***P < 0.001, student t-test. Ns, not significantly different (P>0.5). WT, C57BL/6J, HD, Huntington’s disease. Each dot in the graph represents the data from one animal.

**Supplemental Figure 2. Reduced retinal light responses in R6/1 mice with ages at low flash intensities.**

**(A, B)** Example of ERG traces to flashes with low intensities ( 0.01 and 0.1 cd.s/m^2^) under dark adaptation (A) or at 3.0 cd.s/m^2^ under light adaptation (B) from two groups across ages. **(C, E, G)** Average peak amplitudes of a-wave at scotopic 0.01(C), scotopic 0.1 (E), and photopic 3.0 (G). **(D, F, H)** Average peak amplitudes of b-wave at scotopic 0.01(D), scotopic 0.1 (F), and photopic 3.0 (H). Data are expressed as mean ± SEM;#, P<0.05, ##, P<0.01, ###, P<0.001, two-way ANOVA analysis as groups; **P < 0.01 multiple comparisons by two-way ANOVA. Ns, not significantly different (P>0.5). WT, C57BL/6J, HD, Huntington's disease. Each dot in the graph represents the data from one animal.

**Supplemental Figure 3. Impaired photoreceptor layer in R6/1 retina at middle and peripheral regions.**

**(A, B)** Images of Opsin (green) and DAPI (blue) staining in retina sections from different animal groups at center region across ages. **(C)** The thickness of total retinal layers, the outer nuclei layer (ONL), the inner nuclei layer (INL), and the inner plexiform layer (IPL) at the middle region (1000μm distances away from the optic disk center). **(D)** The thickness of total retinal layers, the outer nuclei layer (ONL), the inner nuclei layer (INL), and the inner plexiform layer (IPL) at the peripheral regions (1500um distances away from the optic disk center). Data are expressed as mean ± SEM; #, P<0.05, two-way ANOVA analysis as groups; *P < 0.05, **P < 0.01, ***P < 0.001, multiple comparisons by two-way ANOVA. WT, C57BL/6J, HD, Huntington’s disease; OD, optic disc; OS, outer segment; ONL, outer nuclei layer; INL, inner nuclei layer; IPL, inner plexiform layer; GCL, ganglion cell layer.
